# Dissociable Effects of Alzheimer's Disease-Related Cognitive Dysfunction and Aging on Functional Brain Network Segregation

**DOI:** 10.1523/JNEUROSCI.0579-23.2023

**Published:** 2023-11-15

**Authors:** Ziwei Zhang, Micaela Y. Chan, Liang Han, Claudia A. Carreno, Ezra Winter-Nelson, Gagan S. Wig, for the Alzheimer's Disease Neuroimaging Initiative (ADNI)

**Affiliations:** ^1^Center for Vital Longevity and School of Behavioral and Brain Sciences, University of Texas at Dallas, Dallas, Texas 75235; ^2^Department of Psychiatry, University of Texas Southwestern Medical Center, Dallas, Texas 75390

**Keywords:** aging, Alzheimer's disease, brain networks, dementia, resting-state fMRI, system segregation

## Abstract

Alzheimer's disease (AD) is associated with changes in large-scale functional brain network organization. Individuals with AD exhibit less segregated resting-state brain networks compared with individuals without dementia. However, declines in brain network segregation are also evident as adult individuals grow older. Determining whether these observations reflect unique or overlapping alterations on the functional connectome of the brain is essential for understanding the impact of AD on network organization and incorporating measures of functional brain network organization toward AD characterization. Relationships between AD dementia severity and participant's age on resting-state brain system segregation were examined in 326 cognitively healthy and 275 cognitively impaired human individuals recruited through the Alzheimer's Disease Neuroimaging Initiative (ADNI) (*N* = 601; age range, 55–96 years; 320 females). Greater dementia severity and increasing age were independently associated with lower brain system segregation. Further, dementia versus age relationships with brain network organization varied according to the processing roles of brain systems and types of network interactions. Aging was associated with alterations to association systems, primarily among within-system relationships. Conversely, dementia severity was associated with alterations that included both association systems and sensory-motor systems and was most prominent among cross-system interactions. Dementia-related network alterations were evident regardless of the presence of cortical amyloid burden, revealing that the measures of functional network organization are unique from this marker of AD-related pathology. Collectively, these observations demonstrate the specific and widespread alterations in the topological organization of large-scale brain networks that accompany AD and highlight functionally dissociable brain network vulnerabilities associated with AD-related cognitive dysfunction versus aging.

**SIGNIFICANCE STATEMENT** Alzheimer's disease (AD)-associated cognitive dysfunction is hypothesized to be a consequence of brain network damage. It is unclear exactly how brain network alterations vary with dementia severity and whether they are distinct from alterations associated with aging. We evaluated functional brain network organization measured at rest among individuals who varied in age and dementia status. AD and aging exerted dissociable impacts on the brain's functional connectome. AD-associated brain network alterations were widespread and involved systems that subserve not only higher-order cognitive operations, but also sensory and motor operations. Notably, AD-related network alterations were independent of amyloid pathology. The research furthers our understanding of AD-related brain dysfunction and motivates refining existing frameworks of dementia characterization with measures of functional network organization.

## Introduction

Adult aging is the greatest risk factor for Alzheimer's disease (AD). AD prevalence doubles every 5 years after the age of 65 years (Hebert et al., 2013). AD is characterized by a wide range of cognitive difficulties, which include deficits in memory and executive function but also sensory and motor processing (Salmon and Bondi, 2009; Albers et al., 2015; Murphy, 2019). The clinical manifestations of the disease are hypothesized to be a consequence of the failure of multiple distributed and functionally specialized brain systems, which are interconnected within a large-scale brain network (Delbeuck et al., 2003; Stam, 2014; Yu et al., 2021). Gaining a deeper understanding of these brain network changes and determining how they differ from the brain network changes that accompany typical aging are critical steps toward establishing the etiology of AD-related brain and cognitive dysfunction.

The functional network organization of the brain can be measured at rest (Petersen and Sporns, 2015). In healthy young adults, the resting-state functional connectome exhibits a modular organization, which is defined by the segregation of large-scale brain systems (Tononi et al., 1994; Sporns and Betzel, 2016; Wig, 2017). Brain system segregation supports the functional specialization of distinct brain regions and individual differences in brain system segregation are related to variability in brain function and cognitive ability (Wig, 2017).

Multiple lines of evidence have demonstrated that resting-state brain system segregation is altered in AD. First, AD patients exhibit fewer modular networks when compared with healthy control participants (Brier et al., 2014a). Second, higher brain system segregation attenuates the effect of AD severity on cognition among both autosomal-dominant AD and sporadic AD patients (Ewers et al., 2021). Finally, among healthy adult individuals, longitudinal changes in brain system segregation are prognostic of dementia independent of AD-related genetic risk, the presence of AD-related pathology (cortical amyloid and CSF tau burden) and structural deterioration (Chan et al., 2021). Collectively, these observations support the idea that the impacts of AD on brain function may not be limited to a small subset of regions or even a single brain system, but rather involve a more extensive set of brain network interactions that span across multiple distributed brain systems, which can be quantified by system segregation.

However, system segregation also changes over the course of healthy adult aging in the absence of AD: brain system segregation declines with increasing adult age (Betzel et al., 2014; Chan et al., 2014; Sala-Llonch et al., 2015; Geerligs et al., 2015b; Han et al. 2018). Further, aging-accompanied changes in brain system segregation are linked to alterations in brain function (Chan et al., 2017), relate to the cognitive changes that accompany adult aging (Chong et al., 2019; Pedersen et al., 2021), and are moderated by environmental exposures during adulthood (Chan et al., 2018, 2021).

Altogether, it is evident that AD and adult aging are associated with reductions in resting-state brain system segregation. What is less clear is whether the functional network changes observed in AD reflect common or unique patterns of reorganization relative to those occurring with normal aging, as comparisons of summary network measures alone can occlude meaningful differences in network topology (Wig, 2017). Untangling the large-scale network correlation patterns associated with AD from those associated with aging would not only advance the ability to discriminate between healthy and pathologic aging but would also accelerate the application of measures of large-scale brain network organization toward AD characterization and staging. Here we set out to resolve the ambiguity by assessing whether there exist distinct relationships between AD dementia severity versus aging on functional brain system segregation and the specific sets of network interactions that comprise the measure.

## Materials and Methods

### Participants

Participants included in the current study were recruited through the Alzheimer's Disease Neuroimaging Initiative (ADNI; for detailed information on the project, see http://www.adni-info.org). Data were collected under ADNI GO, ADNI 2, and ADNI 3 studies, and data were downloaded directly from the ADNI database, all before December 1, 2022 (https://ida.loni.usc.edu/login.jsp?project=ADNI). Written consent was obtained from all participants, and each study was approved by the Institutional Review Board at each participating institution.

The diagnosis status of each participant was assessed at the initial visit using the Wechsler Memory Scale, Mini-Mental State Exam (MMSE; Folstein et al., 1975), clinical dementia rating (CDR; Hughes et al., 1982; Morris, 1993), and the degree of subjective memory concerns (Petersen et al., 2010). The participants categorized as AD were required to meet the NINCDS-ADRDA (National Institute of Neurological and Communicative Disorders and Stroke and the Alzheimer's Disease and Related Disorders Association) criteria for probable AD (McKhann et al., 1984; Petersen et al., 2010). It is important to note that participants enrolled in ADNI included both cognitively healthy subjects and individuals with cognitive impairments. In addition, as enrollment was based on clinical symptoms rather than the presence of specific AD-related neuropathology (Jack et al., 2018), it is possible that a number of cognitively impaired participants may not have AD. However, the majority of participants were categorized as having probable AD based on their set of clinical symptoms (McKhann et al., 1984; Petersen et al., 2010). In keeping with this, the enrichment of the ADNI sample with participants exhibiting AD-related genetic risk and pathologic markers has been confirmed (Aisen et al., 2010; Jagust et al., 2015; Kang et al., 2015) and was also evident in the present report. As such, we refer to participants as having AD-related dementia, but acknowledge here that a small set of participants may not go on to be confirmed for AD. For the detailed inclusion criteria of participant groups, see the general clinical protocols of ADNI (https://adni.loni.usc.edu/methods/documents/).

A total of 783 participants (age range, 55–96 years) were submitted through structural and functional magnetic resonance imaging (fMRI) processing. Participants' data were only included in the final sample if they had available (1) a resting-state fMRI and a structural MRI scan that passed all quality control (QC) procedures for resting-state fMRI and structural MRI processing (described below), (2) CDR score assessed within 6 months of the resting-state fMRI scan (mean, 19 d; range, 0–203 d; SD, 33 d), and (3) demographic (age and self-reported gender) and education information. Variables associated with clinical status (i.e., CDR) were measured in separate clinical sessions. Some participants had multiple resting-state scans available, collected at different dates, and the scan associated with the highest CDR rating was included. Based on the above criteria, a total of 601 participants were included in the final sample (female, *n* = 320; age range, 55–96 years; mean age, 74.70 years; SD, 8.11 years). Additionally, 550 participants in the final sample had scores from the Alzheimer's Disease Assessment Scale—Cognitive Subscale (ADAS-Cog; Rosen et al., 1984) available, each collected in the same session as when their CDR scores were measured.

### Data acquisition and preprocessing

#### Structural MRI.

Under the ADNI protocol, MRI scans were obtained on 3 T scanners in multiple scanning sites using standard scanning protocols. General information about scanning protocols can be found at https://adni.loni.usc.edu/methods/mri-tool/mri-analysis/. The current study included structural MRI scans collected at the same session as resting-state fMRI scans. Each structural MRI was recorded using a 3D T1-weighted magnetization-prepared rapid acquisition gradient echo sequence (TR, 2300 ms; TE, minimum full echo; voxel size, 1 × 1×1 mm).

T1-weighted images were processed using FreeSurfer 6.0 to create cortical surface images. The preprocessing steps included brain extraction, tissue segmentation, generation of white matter and pial surfaces, inflating surfaces to a sphere, and surface shaped-based spherical registration of the participant's native surface to the fsaverage surface (Dale et al., 1999; Fischl et al., 1999a). A single deformation map was created for each participant. The map combined two different deformation maps: one was generated when registering an individual's native surface to FreeSurfer's fsaverage atlas, and the other was generated through registering fsaverage-aligned data to a hybrid left–right fsaverage surface (fs_LR; Van Essen et al., 2012). Each individual's native FreeSurfer-generated output was registered to fs_LR using the single deformation map in a one-step resampling procedure.

#### Amyloid pathology.

A subset of participants had available information related to presence of amyloid pathology. The level of cortical amyloid-β (Aβ) uptake was used to categorize a participant's Aβ pathology. Cortical Aβ was preprocessed and analyzed by ADNI PET core. For general protocols, see https://adni.loni.usc.edu/methods/pet-analysis-method/pet-analysis/. In the current sample, cortical Aβ uptake was measured with either ^18^F-florbetapir or ^18^F-florbetaben imaging tracer. The Aβ uptake values were calculated using the whole cerebellum as the reference region and considered as a continuous measure. In addition, the presence of Aβ pathology was considered categorically, based on cutoff values that were provided by the ADNI PET Core (^18^F-florbetapir, global standardized uptake value ratio (SUVR) > 1.11; ^18^F-florbetaben, global SUVR > 1.08; Landau et al., 2012, 2013). Based on these cutoff values, the sample included 216 Aβ^+^ participants (CDR 0, *N* = 93; CDR 0.5, *N* = 83; CDR 1 and 2, *N* = 40) and 238 Aβ^–^ participants (CDR 0, *N* = 160; CDR 0.5, *N* = 72; CDR 1 and 2, *N* = 6).

#### Resting-state functional MRI.

Resting-state fMRI scans were collected under ADNI GO, ADNI 2, and ADNI 3 studies. Detailed information about scanner protocols can be found at https://adni.loni.usc.edu/methods/documents/mri-protocols/. Functional brain images from ADNI GO that were included used an echoplanar imaging (EPI) blood oxygenation level-dependent (BOLD) sequence (TR, 3000 ms; TE, 30 ms; flip angle, 90°; 48 interleaved axial slices per frame). Each imaging session included one run of a resting-state scan session, and each session consisted of 140 frames. Functional brain images from ADNI 2 that were included used an EPI BOLD sequence (TR, 3000 ms; TE, 30 ms; flip angle, 90°; 48 interleaved axial slices per frame). Each session had one run of resting-state scan session, and each session consisted of 140, 197, or 200 frames, depending on the scanning site. Functional brain images from ADNI 3 that were included used an EPI BOLD sequence (TR, 3000 ms; TE, 30 ms; flip angle, 90°; 48 interleaved axial slices per frame). Each imaging session included one run of resting-state scan session, and each session consisted of 200 frames.

BOLD images (resting state) corresponding to the same session as each of the structural images were processed using a standard fMRI preprocessing pipeline using Nipype 0.8.0. The preprocessing steps included the following: (1) slice-timing correction because of interleaved slice acquisition, using the middle slice as the reference slice; (2) rigid body correction for estimating and correcting head movement between frames; and (3) realignment to the T1-weighted image from the same session. All steps were performed using FSL 6.0, except for realignment between frames and rigid body correction. SPM8 was used for realignment and rigid body correction, as it provided more accurate estimates in our processing stream.

Following standard fMRI preprocessing, additional resting-state functional connectivity (RSFC)-specific processing steps were implemented to reduce spurious variance that was unlikely to reflect neuronal activity in the data. Considerable evidence has shown that older age is associated with greater amounts of head movement (Mowinckel et al., 2012; Van Dijk et al., 2012; Savalia et al., 2017), which has been shown to systematically alter the correlation structure of resting-state signals (Van Dijk et al., 2012; Satterthwaite et al., 2013; Yan et al., 2013; Power et al., 2014, 2015; Zeng et al., 2014). To this end, while a part of the global signal may contain variance related to general levels of arousal and neural activity (Schölvinck et al., 2010; Keller et al., 2013), a major component of the global signal includes spatially nonspecific signal artifacts related to head motion, cardiac signals and breathing (Satterthwaite et al., 2013; Power et al., 2014, 2015, 2017). Removing the global signal thus helps control these known influences of artifact (Yan et al., 2013; Power et al., 2014, 2017). As no method presently exists for denoising known artifactual signals while retaining all remaining “real” signals, the alternate option of retaining the global signal in each participant is likely to result in misestimation of correlations and the resultant network measures. Based on these considerations, we used a series of motion-processing procedures, including global signal regression (GSR) together with data-censoring (“scrubbing”) and signal-processing procedures, as these procedures have been shown to best reduce global and distance-dependent artifacts (Power et al., 2014; Ciric et al., 2017).

RSFC-specific processing steps involved the following steps and order: (1) demeaning and detrending BOLD time series; (2) performing multiple regression of the BOLD data to remove variance associated with whole-brain gray matter signal (GSR), ventricular signal, white matter signal, their derivatives, and the “Friston 24” motion regressors (Friston et al., 1996); (3) removing and interpolating motion-contaminated frames that have frame-by-frame displacement (FD) > 0.3 mm (“scrubbing”; Power et al., 2014); (4) bandpass filtering (0.009–0.08 Hz); and (5) removing the interpolated frames that were used to preserve the time series during regression and bandpass filtering.

Preprocessed resting-state data were registered to the fs_LR (32k) left and right hemisphere surfaces because of improvement in alignment of cortical anatomy in comparison with volume-based registration (Fischl et al., 1999b). Using the transformation matrix and deformation maps generated during preprocessing of the corresponding structural data, volumetric functional data were resampled to the fs_LR surfaces through a one-step transformation. Functional data on fs_LR surfaces were smoothed using a Gaussian smoothing kernel (FWHM, 6 mm).

#### Structural and functional data processing quality control.

All 783 participants' structural and resting-state scans underwent structural and fMRI preprocessing, motion processing, and surface mapping (if possible); 601 participants passed all structural and functional image QC steps. Of the participants who were excluded from subsequent analysis, 157 participants failed the fMRI motion-processing QC (i.e., they had <100 frames remaining after motion scrubbing), 25 participants failed structural processing QC (e.g., because of poor structural skull stripping) or surface-mapping QC.

Individuals who failed motion processing (i.e., had <100 clean frames remaining after motion scrubbing) were not significantly older than those that passed (*t*_(781)_ = 0.19; *p* = 0.84), but exhibited higher CDR scores (*t*_(781)_ = 3.75; *p* < 0.001). As indicated earlier, some individuals had a greater number of resting-state BOLD frames collected. Because our criteria of excluding individuals with high head motion was based on the absolute number of frames remaining (100), individuals who had fewer frames collected may be more likely to be excluded than individuals who have more frames collected. To evaluate whether individuals with higher CDR indeed lost a greater proportion of frames because of head motion, we examined the effect of CDR on the percentage of frame loss relative to the amount originally acquired. Controlling for age, CDR scores were significantly associated with the percentage of frame loss [β = 0.08; *t*_(780)_ = 3.34; partial *r* = 0.12; 95% confidence interval (CI) = 0.03, 0.13; *p* < 0.001]: more demented individuals had a greater percentage of frames lost. This difference in movement-related frame loss is consistent with the observation that before scrubbing, mean head motion during BOLD data acquisition differs as a function of CDR. Critically however, these differences are no longer evident following RSFC motion processing (Table 1). Age was not associated with the percentage of frame loss when controlling for CDR scores (β = 0.001; *t*_(780)_ = 0.69; partial *r* = 0.02; 95% CI = −0.002, 0.003; *p* = 0.49). Individuals who failed structural QCs or surface-mapping QC were younger than individuals who passed preprocessing and surface mapping (*t*_(623)_ = −1.67; *p* = 0.02), but showed no difference in CDR rating (*t*_(623)_ = 0.51; *p* = 0.89). Notably, the number of subjects who failed structural processing QC was substantially small given that most individuals were successfully preprocessed (passed, *N* = 601; failed, *N* = 25), so these age differences should be interpreted with caution.

**Table 1.** ADNI dataset demographic, imaging, health, and AD-related information

Variables	CDR = 0 (*n* = 326)	CDR = 0.5 (*n* = 220)	CDR = 1 and 2 (*n* = 55)	Total (*n* = 601)	*p*
Age, years (SD)	73.96 (7.75)	74.98 (8.60)	77.95 (7.48)	74.70 (8.11)	0.001
Female, *n* (%)	186 (57.06%)	108 (49.09%)	26 (47.27%)	320 (53.24%)	0.12
Education, years (SD)	16.97 (2.17)	16.08 (2.69)	15.42 (2.41)	16.50 (2.45)	<0.001
Pulse rate, beats/min (SD)	64.56 (10.52)	64.13 (10.35)	64.45 (8.92)	64.39 (10.31)	0.76
Respiration rate, breaths/min (SD)	15.94 (2.30)	16.29 (2.45)	16.22 (2.24)	16.09 (2.36)	0.14
APOE4^+^, *n* (%)	90 (27.61%)	76 (34.55%)	32 (58.18%)	198 (32.95%)	<0.001
MMSE, mean score (SD)	29.12 (1.17)	27.37 (2.43)	20.40 (4.70)	26.68 (3.31)	<0.001
Prescrubbing motion, mean FD (SD)	0.16 (0.06)	0.16 (0.06)	0.19 (0.06)	0.16 (0.06)	0.001
Postscrubbing motion, mean FD (SD)	0.12 (0.03)	0.12 (0.03)	0.13 (0.03)	0.13 (0.03)	0.61

The mean (SD) and counts (%) of numerical and categorical variables are shown for each CDR group and the entire sample. Statistical differences among the CDR groups were calculated using one-way ANOVA for continuous variables and χ^2^ tests for categorical variables (*p* values reported). Participants included were identified with their maximum CDR level during the study. The resting-state scans included were acquired within 6 months from their clinical sessions when CDR was measured. MMSE scores were measured in the same clinical session as CDR score evaluation, available for 598 participants. Vital signs including pulse and respiration rate were measured at the same session as CDR, available for 591 participants. APOE4 status available for 517 participants: participants with at least one copy of APOE4 were categorized as APOE4^+^.

### Brain network construction

Following surface mapping, a functional correlation matrix was constructed for each participant. To control for the variable number of clean frames across participants, for each participant, the first 100 clean frames were used in brain network analyses. The correlation matrix was generated with 441 surface-based nodes that were defined with boundary-based analyses (Chan et al., 2014; Wig et al., 2014). Nodes were generated with the following steps: (1) identifying putative area centers that were the local minima of a previously published RSFC-boundary map (Cohen et al., 2008; Wig et al., 2014); (2) creating disks with a radius of 3 mm around the identified area centers to avoid area borders that may exhibit more variance between individuals. All vertices within a node disk were identified based on their spatial overlap with an a priori vertex-wise community map in the same fs_LR space (Power et al., 2011), where each disk was labeled with a functional system based on a winner-take-all approach.

The BOLD time series of all vertices within each node were averaged to obtain the mean time series of the node. A correlation matrix (brain network) was constructed by computing the pairwise Fisher's *z*-transformed Pearson's correlation of each of the 441 nodes (Zar, 1996). Because GSR may introduce spurious negative correlations (Murphy et al., 2009), negative correlations were excluded from the correlation matrix (i.e., setting all negative values to zero). Edge density thresholding was not applied to the correlation matrix, as calculating brain system segregation (described below) does not require a sparse network matrix.

### System segregation calculation

Brain system segregation is defined as a measure of how segregated each functional brain system is from each other (Chan et al., 2014; Wig, 2017). For each participant, brain system segregation was calculated as the difference between the mean within-system correlation of all systems and the mean between-system correlation of all systems in relation to the mean within-system correlation.

The general formula of brain system segregation is as described in the previous report (Chan et al., 2021), as follows:

$$\text{Brain System Segregation}=\frac{\frac{\sum_{w}^{W}Z_{w}}{W}-\frac{\sum_{b}^{B}Z_{b}}{B}}{\frac{\sum_{w}^{W}Z_{w}}{W}}.$$

In the above formula, $Z_{w}$ represents the mean within-system correlation and $Z_{b}$ represents the mean between-system correlation. W is the number of within-system correlations across all brain systems, and B is the number of between-system correlations across all brain systems. Positive or higher segregation values reflect higher within-system correlations compared with between-system correlations, indicating higher separation (or “differentiation”) of brain systems. When a brain system is disconnected from all other systems, it obtains the maximal segregation value of 1. Conversely, negative or lower values reflect lower within-system correlations in relation to between-system correlations, indicating lower separation (or differentiation) of brain systems.

**Figure 1.**
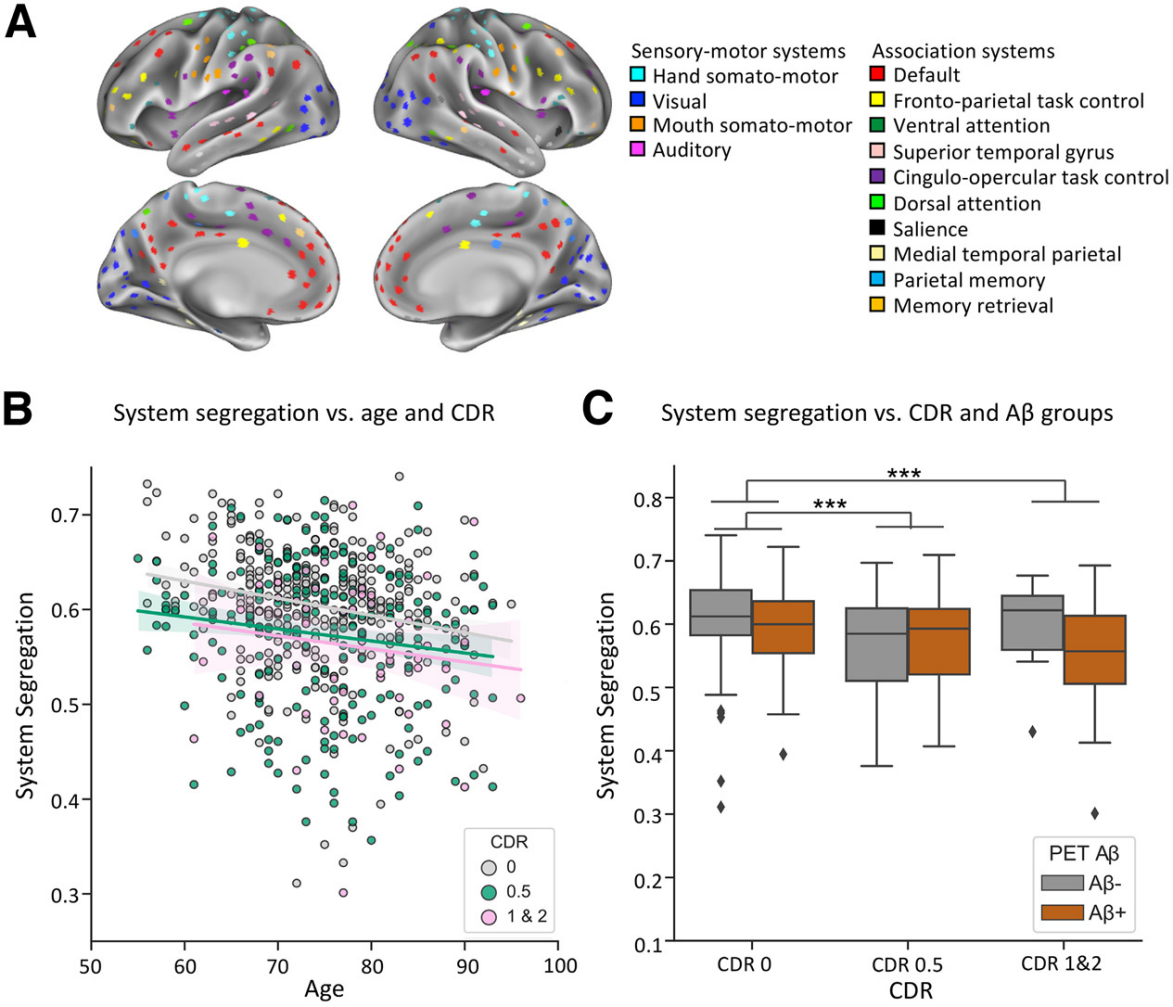
Resting-state brain system segregation decreases as a function of both age and dementia severity. ***A***, Resting-state functional brain networks of each participant were constructed based on nodes (Chan et al., 2014) that are labeled by corresponding functional systems (Power et al., 2011). ***B***, Brain system segregation is plotted for each individual as a function of their age and CDR rating (dementia severity). Older adult individuals exhibit lower brain system segregation than younger adults, reflecting a disrupted modular organization. This relationship is evident across all levels of CDR scores (*p* < 0.001). Irrespective of age, increasing dementia severity (measured with the participant's CDR score) is associated with lower brain system segregation (*p* < 0.001). For the scatterplot, colored lines reflect the linear regression between age and system segregation as a function of CDR ratings. The shading of each color line depicts the 95% confidence interval for the regression estimates between age and system segregation. ***C***, Brain system segregation is plotted for each individual as a function of their CDR rating (dementia severity) and PET Aβ status. Higher dementia severity is associated with lower brain system segregation, which remains evident across both the Aβ^–^ and Aβ^+^ groups of individuals [note that there are very few individuals that are classified as Aβ^–^ who have CDR ratings of either 1 or 2 (*N* = 6)]. Asterisks between bars indicate a significant difference in brain system segregation revealed by *post hoc t* tests; ***p < 0.001, corrected for multiple comparisons.

The segregation of specific types of brain systems (i.e., sensory-motor system segregation, association system segregation; Fig. 1*A*, system categorization) were also calculated for each participant. Sensory-motor systems are primarily involved in processing sensory inputs and motor outputs. Association systems are involved in more “higher-order” integration of information (Mesulam, 1990; Petersen and Posner, 2012). For segregation of specific system types, within-system and between-system correlations are pooled from specific functional systems. First, for each functional system that is classified in a given system type (Fig. 1*A*), the mean within-system correlation ($W_{s}$; average correlations between nodes that belong to the same system S) and the mean between-system correlation ($B_{s}$, average correlations between a single system, S, to all other systems) is calculated. Then, system type-specific segregation is calculated as the difference between the grand mean of mean within-system correlations $\bar{W_{s}}$ and the grand mean of mean between-system correlations $\bar{B}$, in relation to the grand mean of $\bar{W_{s}}$ (Chan et al., 2014).

### Statistical analysis

The present study used a cross-sectional design to determine the relationship between age and dementia severity on functional brain network organization. Multiple linear regression models were used to examine the effects of age and CDR scores on brain system segregation. CDR rating was coded as a continuous variable to model dementia severity on a linear scale for the primary analyses. A subset of participants had available a measure of cortical Aβ deposition or their apolipoprotein ε4 allele (APOE4) status, which were each treated as independent variables in separate multiple linear regression models. Additional variables included participant's gender, head motion (i.e., postscrubbing mean FD) and years of education; all reported linear regression models controlled for these covariates. A subset of participants had available measures of pulse rate and respiration rate (Table 1, caption); these latter variables were also included as covariates in secondary analyses.

For all analyses, including the effect of imaging sites as an additional covariate did not alter the conclusions presented throughout the report.

Higher-order interaction terms were included in linear regression models. In several cases, there was an absence of a significant effect of the interaction terms, and the total variance explained by the model was either comparable to or lower than a corresponding model in which the interaction terms were removed. Considering this, together with the demonstration that including all possible interaction terms can lead to increasing rates of false negatives and false positives of other effects included in the model (Quinn and Keough, 2002; Engqvist, 2005; Huitema, 2011), under this scenario we removed these higher-order terms and recalculated the models, limiting the estimation to main effects of the variables.

Where applicable, multiple-comparison correction on *post hoc* tests was performed with a Bonferroni correction.

To examine relationships within and across specific systems, for each participant, block matrices were computed from their node-by-node RSFC matrix, based on predefined system labels (Power et al., 2011). For each system, within-system blocks were computed as the average correlation across relationships among nodes in the same system, and between-system blocks were computed as the average correlation across relationships among nodes between pairs of different systems. Given that the distributions of node-by-node correlations in some blocks can be skewed, statistical significance was evaluated using a permutation test (*N* = 1000) by randomly shuffling CDR labels across participants. The permutation test was computed by controlling for all covariates in the original model. Blocks that exhibited statistically significant regression coefficients were visualized [*p* < 0.05 uncorrected and false discovery rate (FDR) corrected].

We also directly tested whether the effects of age and CDR on system segregation varied according to system type using linear mixed-effects models. Three separate models were used to test the interactions between age and system type, CDR and system type and their three-way interactions. System type was considered as a within-subject effect, and other variables were included as between-subject effects. The effect of age was controlled in the model that tested the interaction between CDR and system type, while the effect of CDR was controlled in the model that tested the interaction between age and system type. Parallel analyses were performed to evaluate differences in mean network interactions for within-system network interactions versus between-system network interactions.

### Software availability

Multiple linear regression models and linear mixed-effects models were performed in Python 3.8 using statsmodels (version 0.12.0). Data visualization was conducted in Python 3.8 using seaborn (version 0.11.1). Block-level matrix comparisons were conducted in MATLAB R2020b using in-house scripts. Visualization of nodes on cortical surfaces were generated using Connectome Workbench (version 1.4.2).

### Code availability

The code of multiple linear regression and linear mixed-effects models is available at https://github.com/ziweizhang2405/python_statsmodel_brain_network. The calculation of brain system segregation is available at https://gitlab.com/wiglab/system-segregation-and-graph-tools.

### Data availability

Data used in the study are available to investigators on request and approval from the ADNI Data and Publications Committee. Instructions for making a request can be found at https://adni.loni.usc.edu/data-samples/access-data/.

## Results

### Participants characteristics

Participant's ages ranged from 55 to 96 years at the time of their fMRI data acquisition (*N* = 601; 320 females; Table 1, additional participant characteristics). Individuals designated as cognitively normal, mildly cognitive impaired (MCI), and demented were all included in the sample. Given that the CDR system is more sensitive and reliable in measuring cognitive dysfunction than alternate cohort labels and rating systems (Balsis et al., 2015), dementia severity was defined by the participant's CDR scores, which were measured close in time to their brain imaging session (mean time, 19 d; time range, 0–203 d; Hughes et al., 1982; Morris, 1993). As the number of participants with a CDR status of 1 and 2 (corresponding to mild and moderate dementia, respectively) were substantially fewer than those with scores of 0 and 0.5, these former two groups were combined. Age varied across CDR scores thus allowing us to untangle the relationships among age, dementia severity, and patterns of brain network organization.

### Age and dementia severity independently relate to brain system segregation

Brain networks were constructed for each individual using a predefined atlas of nodes and functional system assignments (Fig. 1*A*; Power et al., 2011; Chan et al., 2014). To determine the relationship between age and dementia severity on brain system segregation, we examined the effects of age and CDR scores on cortex-wide brain system segregation, controlling for participant's gender, head motion (i.e., postscrubbing mean FD) and years of education (all reported linear regression models controlled for these covariates).

First, a statistical model demonstrated the absence of a significant interaction between participant age and CDR status on brain system segregation (age × CDR: β = 0.0007; 95% CI = −0.001, 0.003; *t*_(594)_ = 0.60; partial *r* = 0.02; *p* = 0.55).

A separate multiple linear regression model that included the main effects of age and CDR status explained a significant amount of variance in brain system segregation (*R*^2^ = 0.13; adjusted *R*^2^ = 0.12; *F*_(5,595)_ = 16.98; *p* < 0.001). As depicted in Figure 1*B*, age exhibited a significant relationship with brain system segregation (β = −0.002; 95% CI = −0.002, −0.0001; *t*_(595)_ = −4.18; partial *r* = −0.17; *p* < 0.001); older adults exhibited less segregated functional brain networks than their younger counterparts. Notably, CDR status also exhibited a significant relationship with brain system segregation (β = −0.04; 95% CI = −0.06, −0.03; *t*_(595)_ = −4.90; partial *r* = −0.20; *p* < 0.001); greater dementia severity was associated with lesser brain network segregation, independent of the variance explained by age.

In the preceding analyses, CDR status was included as a continuous variable to examine the relationship between increasing dementia severity and brain network organization. Treating CDR status as a categorical variable yielded similar results. The model explained a significant amount of variance in brain system segregation (*R*^2^ = 0.13; adjusted *R*^2^ = 0.12; *F*_(6,594)_ = 14.74; *p* < 0.001). Patients with CDRs of 1 and 2 did not show a difference in cortex-wide system segregation from individuals with CDR = 0.5 (*t*_(273)_ = 1.01; *p* = 0.94), but individuals with CDRs of 0.5 (*t*_(544)_ = 5.17; *p* < 0.001, corrected for multiple comparisons) and CDRs of 1 and 2 (*t*_(379)_ = 4.46; *p* < 0.001, corrected for multiple comparisons) exhibited significantly lower cortex-wide system segregation than cognitively normal participants (i.e., CDR = 0).

To further account for potential sources of non-neuronal variability across participants, participant's heart rate and respiration were included in statistical models as additional covariates (a subset of participants had these measures available; *N* = 591). Similar to the initial findings, the multiple linear regression model explained a significant amount of variance in brain system segregation (*R*^2^ = 0.13; adjusted *R*^2^ = 0.12; *F*_(7,583)_ = 12.58; *p* < 0.001). The main effects of CDR status and age remained significant (CDR: β = −0.04; 95% CI = −0.06, −0.02; *t*_(583)_ = −4.69; partial *r* = 0.19; *p* < 0.001; age: β = −0.001; 95% CI = −0.002, −0.001; *t*_(583)_ = −4.25; partial *r* = 0.17; *p* < 0.001). Pulse rate and respiration rate were not significantly related to brain system segregation (pulse: β = −0.0005; 95% CI = −0.001, 0.0001; *t*_(583)_ = −1.86; partial *r* = 0.08; *p* = 0.06; respiration: β = −0.0008; 95% CI = −0.003, 0.002; *t*_(583)_ = −0.67; partial *r* = 0.03; *p* = 0.51).

Given that the ADNI participant cohort is tailored toward the presence of AD, it is important to consider the extent to which the relationship between dementia severity and brain network organization are related to the presence of AD-related pathology. A subset of participants had available PET-based measures of cortical Aβ; the mean cortical Aβ SUVR was included together with participants age and CDR scores, to evaluate their relations to brain system segregation. The first statistical model included higher-order interactions and demonstrated an absence of a significant three-way interaction among cortical Aβ deposition, age, and CDR status on brain system segregation (age × CDR × Aβ SUVR: β = 0.007; 95% CI = −0.005, 0.02; *t*_(443)_ = 1.15; partial *r* = 0.05; *p* = 0.25). In addition, there were no significant two-way interactions between any of the independent variables on brain system segregation (all *p* values > 0.10). A separate multiple linear regression model that included the main effects of cortical Aβ deposition, age, and CDR status explained a significant amount of variance in brain system segregation (*R*^2^ = 0.13; adjusted *R*^2^ = 0.11; *F*_(6,447)_ = 10.66, *p* < 0.001). The main effects of CDR and age were significant (CDR: β = −0.05; 95% CI = −0.07, −0.03; *t*_(447)_ = −4.54; partial *r* = −0.21; *p* < 0.001; age: β = −0.001; 95% CI = −0.002, −0.0001; *t*_(447)_ = −3.06; partial *r* = −0.14; *p* = 0.002), while cortical Aβ deposition was not significantly associated with brain system segregation (β = 0.01; 95% CI = −0.02, 0.04; *t*_(447)_ = 0.70; partial *r* = 0.03; *p* = 0.49). Stratifying individuals based on established cut points of PET Aβ SUVR did not alter the significant effects of CDR and age (*R*^2^ = 0.13; adjusted *R*^2^ = 0.11; *F*_(6,447)_ = 10.60; *p* < 0.001; CDR: β = −0.04; 95% CI = −0.07, −0.02; *t*_(447)_ = −4.34; partial *r* = −0.20; *p* < 0.001; age: β = −0.001; 95% CI = −0.002, −0.0001; *t*_(447)_ = −2.92; partial *r* = −0.14; *p* = 0.004; cortical Aβ (Aβ^+^ vs Aβ^–^): β = −0.003; 95% CI = −0.02, 0.01; *t*_(447)_ = −0.38; partial *r* = −0.02; *p* = 0.70). Figure 1*C* depicts these relationships whereby participants were categorized as cortical Aβ^+^ versus Aβ^–^ (based on cutoffs established by the ADNI PET Core; Landau et al., 2012, 2013).

Likewise, a subset of participants had their APOE4 status avaliable. The absence or presence of the APOE ε4 allele (i.e., at least one copy of the APOE ε4 allele) was included together with participant ages and CDR scores to evaluate their relations to brain system segregation. The first linear regression model included higher-order interactions and indicated an absence of a significant three-way interaction among CDR, age, and APOE4 status (CDR × age × APOE4 status: β = 0.001; 95% CI = −0.001, 0.004; *t*_(506)_ = 0.89; partial *r* = 0.04; *p* = 0.38). In addition, there were no significant two-way interactions between any of the independent variables on brain system segregation (all *p* values > 0.30). A separate multiple linear regression model that included the main effects of age, CDR status, and APOE4 status explained a significant amount of variance in brain system segregation (*R*^2^ = 0.11; adjusted *R*^2^ = 0.10; *F*_(6,510)_ = 10.13; *p* < 0.001). The effects of CDR and age remained significant (CDR: β = −0.04; 95% CI = −0.05, −0.02; *t*_(510)_ = −3.79; partial *r* = −0.17; *p* < 0.001; age: β = −0.001; 95% CI = −0.002, −0.0001; *t*_(510)_ = −2.92; partial *r* = −0.13; *p* = 0.004), while APOE4 status was not significantly associated with brain system segregation (β = 0.006; 95% CI = −0.001, 0.01; *t*_(510)_ = 1.74; partial *r* = 0.08; *p* = 0.08).

Collectively, the observations indicated that adult aging and dementia severity uniquely relate with the segregation of large-scale resting-state brain systems. Importantly, the difference in the segregation of brain systems between cognitively healthy and demented individuals cannot be fully explained by the presence of amyloid burden or AD genetic risk and does not seem to be moderated by these factors. Advanced aging and the presence of severe cognitive impairment are each associated with less segregated resting-state network organization, possibly because of the reorganization of distinct parts of the brain network.

### Age and dementia severity are associated with modified interactions among distinct functional systems

**Figure 2.**
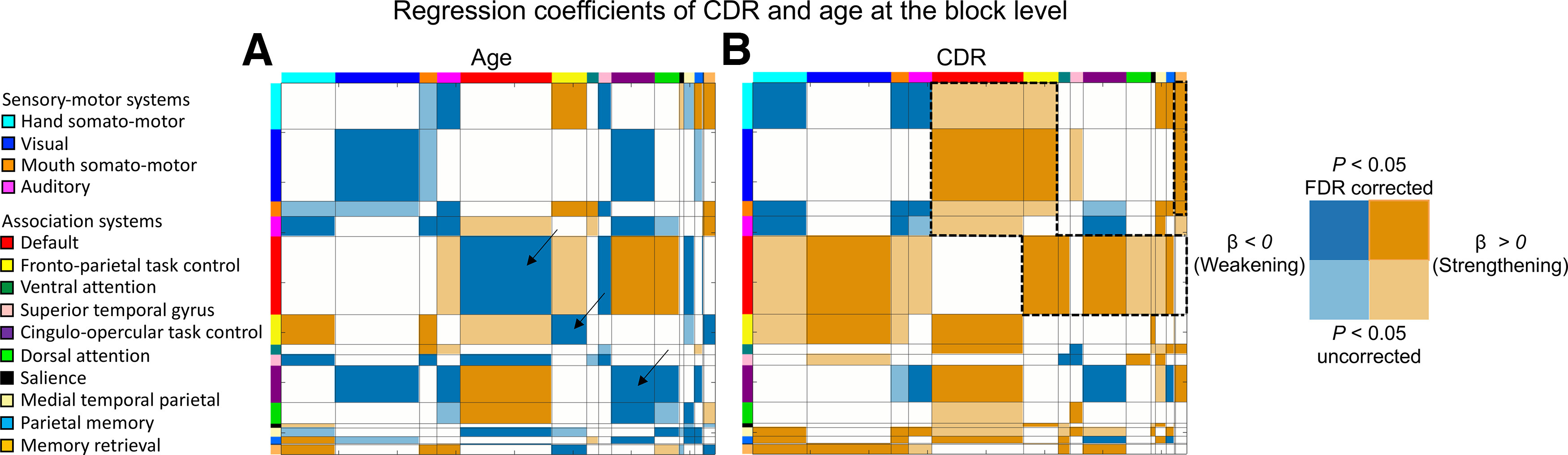
System-wide relationships vary in relation to increasing dementia severity and age. ***A***, ***B***, Matrices depict significant relationships between a participant's age (***A***) and CDR scores (***B***), while controlling for the alternate independent measure, on mean correlations between sets of nodes within and across individual brain systems (block level). Age-accompanied alterations are most prominent among within-system relationships (on-diagonal), particularly reflecting a weakening of within-system relationships among association systems [e.g., default system, frontoparietal task control system, cingulo-opercular task control system (black arrows)]. In contrast, higher CDR is accompanied by widespread alterations that are most prominent among between-system relationships (off-diagonal) and include both sensory-motor and association systems. These alterations are most notable in relationships involving the default system, fronto-parietal task control system and memory retrieval system with other association and sensory-motor systems (black dotted borders). Each matrix depicts significant regression coefficients (darker shading: *p* < 0.05, FDR corrected; lighter shading: *p* < 0.05, uncorrected).

The measure of brain system segregation aggregates relationships across all examined brain systems. It is possible that adult aging and AD dementia are related to alterations in unique brain systems and/or types of functional relationships that are not captured by the summary measure of brain network organization (Wig, 2017). This possibility was confirmed by examining matrices depicted in Figure 2, which revealed the mean system-to-system relationships varying with age and CDR (while controlling for the alternate measure, in addition to controlling for gender, postscrubbing mean FD, and years of education). Increasing age was most prominently associated with weakening of relationships within several brain systems including the frontoparietal task control, cingulo-opercular task control, default, and visual and auditory systems. Increasing age was also associated with the strengthening of relationships between the default and dorsal attention system and between the default and the cingulo-opercular task control system (Fig. 2*A*). Increasing dementia severity was associated with a distinct set of alterations relative to aging. These alterations were prominent among cross-system interactions across the brain. In particular, there was a strengthening of relationships between the default system with several major brain systems, including the visual system, frontoparietal task control system, ventral attention system and the cingulo-opercular task control system (Fig. 2*B*). Higher dementia severity was also associated with stronger relationships between the frontoparietal task control system and multiple major brain systems, including the visual system and the default mode system and between the memory retrieval system and multiple sensory-motor systems. Moreover, increasing dementia severity was also associated with the weakening of relationships within the hand somato-motor system, the mouth somato-motor system, and the cingulo-opercular task control system. Together, it appears that adult aging particularly modifies functional relationships among systems involved in more higher-order integration of information (association systems), while dementia severity relates to functional relationship differences across both association and sensory-motor systems, which are involved in processing sensory inputs and motor outputs (Fig. 1*A*, specific distinctions; Mesulam, 1990; Petersen and Posner, 2012).

The apparent distinctions in functional system vulnerability were tested directly by evaluating differences in brain system segregation for sensory-motor systems versus system segregation for association systems. Specifically, we tested whether the effects of age and CDR on system segregation varied according to system type, whereby system type referred to categorization of sensory-motor versus association systems (Fig. 1*A*).

A linear mixed-effects model included a three-way interaction among age, CDR, and system type on system segregation indicated no significant three-way interaction between the three variables (age × CDR × system type: β < −0.001; 95% CI = −0.001, 0.001; *t*_(597)_ = −0.61; partial *r* = −0.02; *p* = 0.54).

**Figure 3.**
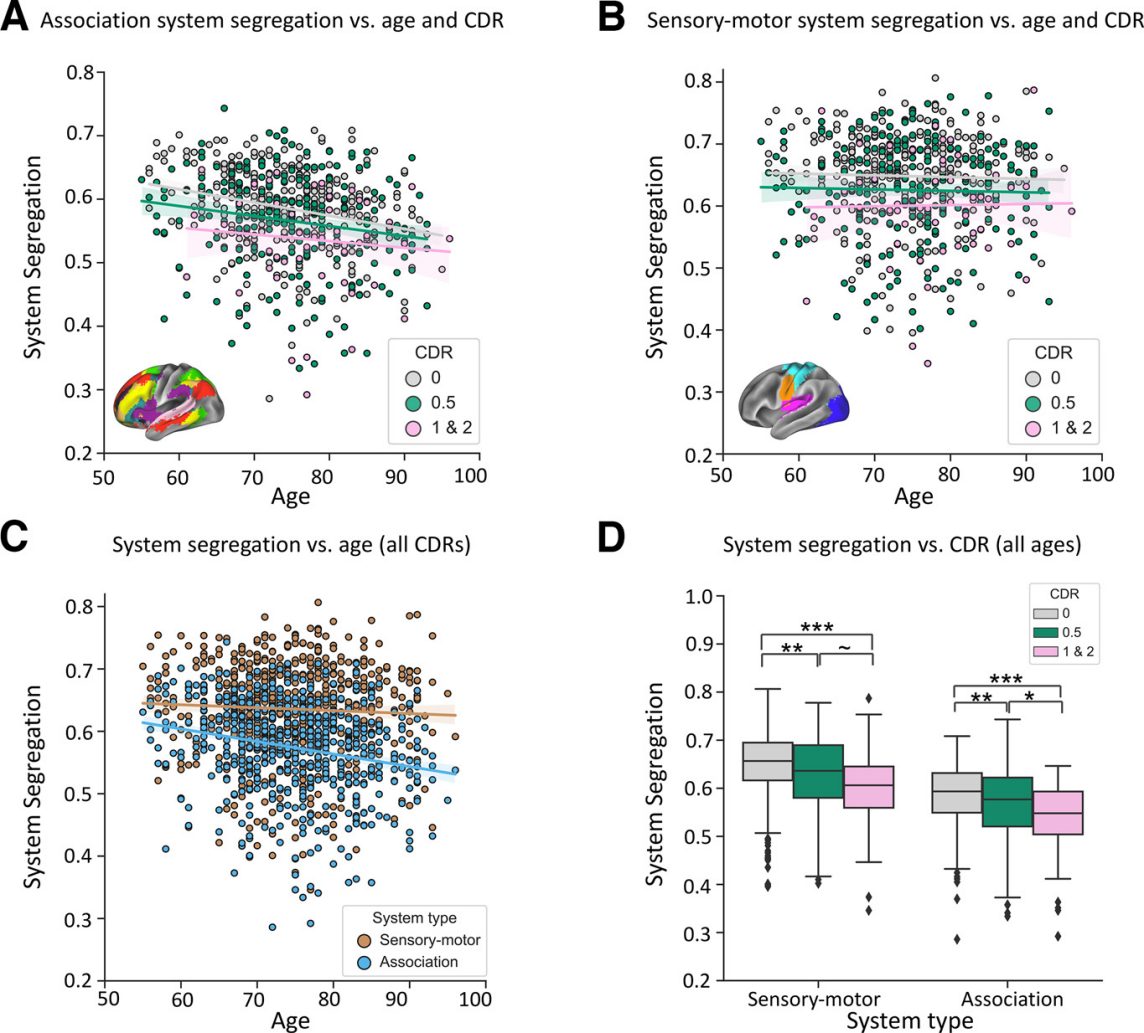
Dementia severity and age exhibit different relationships with the segregation of sensory-motor system versus association system. ***A***, Association system segregation is plotted for every individual as a function of their age and CDR score. Increasing adult age is associated with lower association system segregation, regardless of CDR score. Similarly, increasing CDR score is associated with lower association system segregation, regardless of age. ***B***, Sensory-motor system segregation is plotted for every individual as a function of their age and CDR score. Increasing dementia severity (CDR) is associated with lower sensory-motor system segregation, regardless of age. In contrast to association system segregation, sensory-motor system segregation is not related to age. For the scatterplots, colored lines reflect the linear regression between age and system segregation as a function of CDR scores. The shading of the colored lines depicts the 95% confidence interval for the regression estimates between age and system segregation. ***C***, With increasing age, association system segregation decreases more than sensory-motor system segregation. Colored lines reflect the linear regression between age and system segregation as a function of system types. The shading of the colored lines depicts the 95% confidence interval for the regression estimates between age and system segregation for both types of systems. ***D***, Greater dementia severity (higher CDR score) is associated with lower segregation of both association system and sensory-motor systems. For the boxplot, the colored boxes denote the quartiles of the system segregation values of each CDR group. The whiskers include values that fall outside of the interquartile range with individual dots denoting the outliers of each CDR group. Asterisks between bars indicate a significant difference in brain system segregation revealed by *post hoc t* tests; *p < 0.05, **p < 0.01, ***p < 0.001, corrected for multiple comparisons. The group difference that did not survive Bonferroni correction is also denoted (∼*p* < 0.05, uncorrected).

A second linear mixed-effects model predicting system segregation revealed a significant interaction between age and system type, controlling for CDR and other covariates (conditional *R*^2^ = 0.68; marginal *R*^2^ = 0.21; age × system type: β = 0.001; 95% CI = 0.0001, 0.001; *t*_(599)_ = 4.68; partial *r* = 0.19; *p* < 0.001). As shown in Figure 3, increasing age was associated with decreasing system segregation of association systems (β = −0.002; 95% CI = −0.003, −0.001; *t*_(595)_ = −5.18; partial *r* = −0.21; *p* < 0.001, corrected for multiple comparisons; Fig. 3*A*,*C*), but not of sensory-motor systems (β = −0.0004; 95% CI = −0.001, 0.0001; *t*_(595)_ = −1.09; partial *r* = −0.04; *p* = 0.98; Fig. 3*B*,*C*). In contrast, an additional linear mixed-effects model predicting system segregation revealed no significant interaction between CDR and system type, controlling for age and other covariates (CDR × system type*:* β < 0.001; 95% CI = −0.008, 0.008; *t*_(599)_ = 0.07; partial *r* = 0.02; *p* = 0.94). As shown in Figure 3, increasing dementia severity was associated with decreasing brain system segregation for both association systems (β = −0.04; 95% CI = −0.06, −0.02; *t*_(595)_ = −4.64; partial *r* = −0.19; *p* < 0.001, corrected for multiple comparisons; Fig. 3*A*,*D*) and sensory-motor systems (β = −0.04; 95% CI = −0.06, −0.03; *t*_(595)_ = −4.50; partial *r* = −0.18; *p* < 0.001, corrected for multiple comparisons; Fig. 3*B*,*D*). *Post hoc t* tests demonstrated that individuals with CDR of 0.5 and CDR of 1 and 2 showed lower association system segregation than individuals with CDR = 0 (CDR = 0.5 vs CDR = 0: *t*_(544)_ = 3.42; *p* < 0.001; CDR = 1 and 2 vs CDR = 0: *t*_(379)_ = 5.20; *p* < 0.001; both values were corrected for multiple comparisons). Individuals with CDRs of 1 and 2 also showed lower association system segregation than individuals with CDRs of 0.5 (*t*_(273)_ = 2.50; *p* = 0.04, corrected for multiple comparisons). Similarly, individuals with CDRs of 0.5 and CDRs of 1 and 2 showed lower sensory-motor system segregation than individuals with CDRs of 0 (CDR = 0.5 vs CDR = 0: *t*_(544)_ = 3.44; *p* = 0.002; CDR = 1 and 2 vs CDR = 0: *t*_(379)_ = 4.40, *p* < 0.001; both were corrected for multiple comparisons). Individuals with CDRs of 1 and 2 showed lower sensory-motor system segregation than individuals with CDRs of 0.5 (*t*_(273)_ = 1.99; *p* = 0.048, uncorrected), confirming that aging and dementia relate to alterations in functional relationships among nonequivalent types of brain systems.

### Impacts of dementia severity and age vary for different types of network interactions

In the matrices depicted in Figure 2, it is evident that aging and dementia severity may be dissociable not only according to processing roles of functional systems, but also by the types of network interactions. Specifically, age appears to be more related to interactions among nodes within brain systems, while dementia severity appears to be more related to the interactions spanning across (between) brain systems. We tested the hypothesis of this dissociation directly.

A linear mixed-effects model including a three-way interaction among age, CDR, and network interaction on system segregation indicated no significant three-way interaction among the three variables (age × CDR × network interaction type: β < 0.001; 95% CI = −0.0003, 0.001; *t*_(597)_ = 0.81; partial *r* = 0.04; *p* = 0.42).

**Figure 4.**
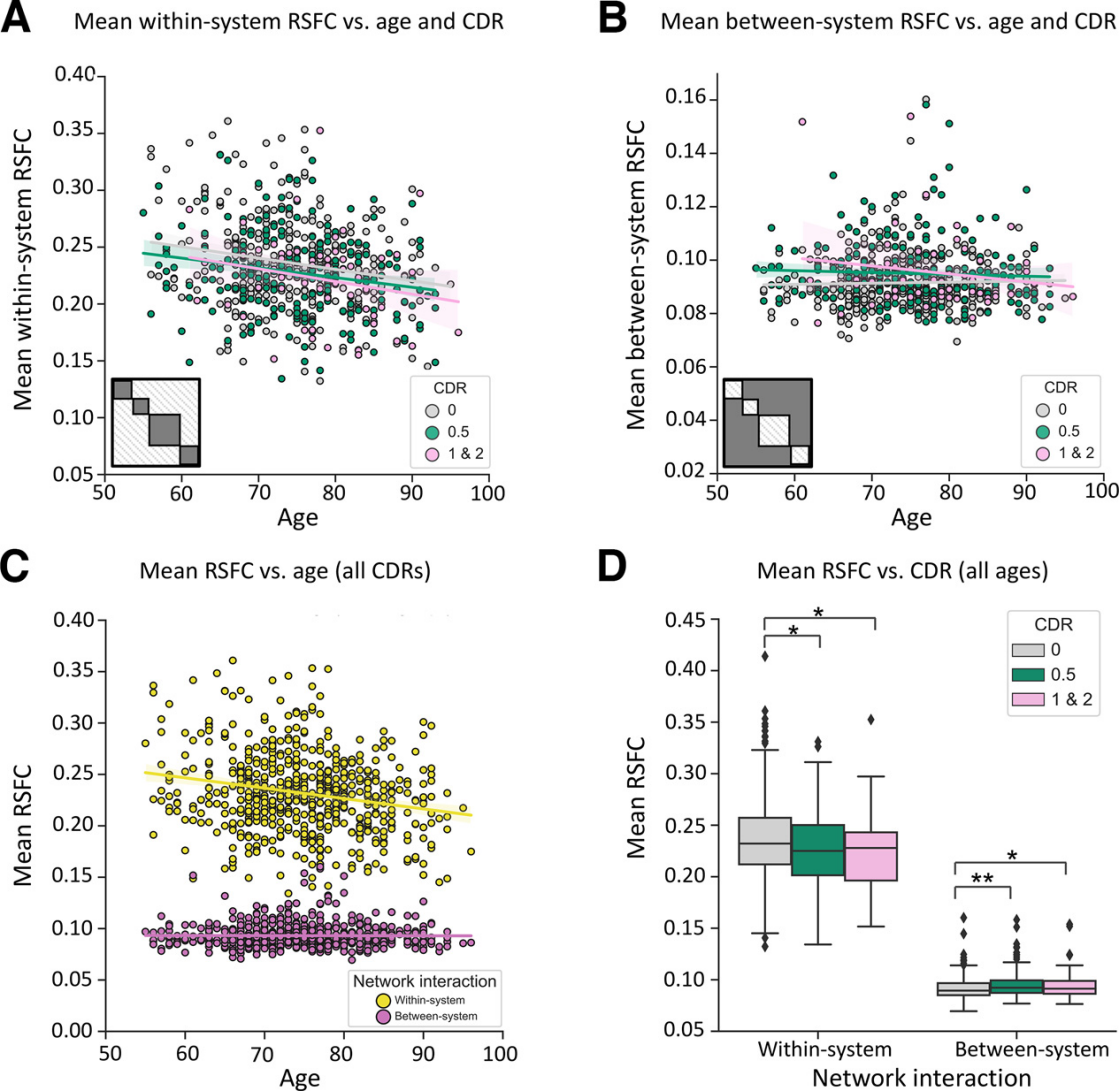
Dementia severity and age are associated with alterations in distinct types of network interactions. ***A***, Within-system network interactions averaged across all systems are plotted as a function of the participant's age and CDR score. Increasing adult age is related to lower mean within-system interactions, regardless of CDR score. CDR is less related to differences in within-system interactions. ***B***, Between-system network interactions averaged across all systems are plotted as a function of the participant's age and CDR score. Increasing dementia severity (CDR) is associated with higher mean between-system interactions, regardless of age. In contrast to CDR, age is not related to differences in between-system interactions. For the scatterplots, colored lines indicate the linear regression between age and network interactions (i.e., within-system and between-system interactions). ***C***, Older age is associated with decreasing within-system interactions. Colored lines depict the linear regression between age and mean network interactions as a function of network interaction types. ***D***, Greater dementia severity is associated with increasing between-system interactions and to a lesser extent, decreasing within-system interactions (see text for details). For the boxplot, the colored boxes denote the quartiles of mean network interactions of each CDR group. The whiskers include values that fall outside of the interquartile range with individual dots denoting the outliers of each CDR group. Asterisks between bars indicates a significant difference in network interactions revealed by *post hoc t* tests; *p < 0.05, **p < 0.01, corrected for multiple comparisons. Nonsignificant comparisons are not denoted. In ***A***, ***B***, and ***C***, the shading of the colored lines indicates the 95% confidence intervals for the regression estimates between age and network interactions as a function of CDR score or types of network interactions.

The second linear mixed-effects model revealed a significant interaction between age and network interaction type, controlling for CDR and other covariates (conditional *R*^2^ = 0.88; marginal *R*^2^ = 0.86; age × network interaction type: β < −0.001; 95% CI = −0.001, −0.0003; *t*_(599)_ = −5.41; partial *r* = −0.22; *p* < 0.001, corrected for multiple comparisons). There was a significant effect of age on mean within-system network interactions (β < −0.001; 95% CI = −0.001, −0.0001; *t*_(595)_ = −4.60; partial *r* = −0.19; *p* < 0.001, corrected for multiple comparisons; Fig. 4*A*), which was not evident when comparing age with between-system interactions (β < −0.001; 95% CI = −0.0001, 0.0001; *t*_(595)_ = −0.75; partial *r* = −0.03; *p* = 0.98; Fig. 4*B*). This distinction is summarized in Figure 4*C*. A separate linear mixed-effects model also indicated a significant interaction between CDR and network interaction type, controlling for age and other covariates (conditional *R*^2^ = 0.88; marginal *R*^2^ = 0.85; CDR × network interaction type*:* β = −0.01; 95% CI = −0.01, −0.006; *t*_(599)_ = −4.51; partial *r* = −0.17; *p* < 0.001). CDR was significantly associated with stronger mean between-system network interactions (β = 0.005; 95% CI = 0.002, 0.008; *t*_(595)_ = 3.27; partial *r* = −0.13; *p* = 0.005, corrected for multiple comparisons; Fig. 4*B*), and exhibited a weaker (and negative) relationship with mean within-system network interactions (β = −0.01; 95% CI = −0.02, 0.002; *t*_(595)_ = −2.45; partial *r* = −0.10; *p* = 0.06; Fig. 4*A*). This distinction is summarized in Figure 4*D*. Aging and AD dementia relate with functional relationships among nodes that show distinct types of network interactions.

### Evaluation of relationships between dementia severity and brain system segregation using alternate measures of cognitive dysfunction

The CDR system is a clinically sensitive and reliable tool for measuring cognitive dysfunction (Perneczky et al., 2006; Fagundes Chaves et al., 2007; Balsis et al., 2015). However, to verify that the relationships among age, dementia severity, and system segregation extend to other assessments of cognitive dysfunction, we evaluated participant's ADAS-Cog scores. ADAS-Cog is a continuous measure that incorporates multiple cognitive tasks including memory, attention, and language function. Controlling for age, participant's ADAS-Cog scores were significantly related to brain system segregation (β = −0.001; 95% CI = −0.001, −0.0001; *t*_(544)_ = −2.53; partial *r* = −0.11; *p* = 0.01). Higher scores, corresponding to worse cognitive function, were associated with lower system segregation. Likewise, paralleling the findings with CDR scores, ADAS-cog scores were negatively associated with sensory-motor system segregation (β = −0.001; 95% CI = −0.002, −0.0001; *t*_(544)_ = −3.45; partial *r* = −0.15; *p* = 0.001, corrected for multiple comparisons), association system segregation (β = −0.001; 95% CI = −0.002, −0.0001; *t*_(544)_ = −3.10; partial *r* = −0.13; *p* = 0.004, corrected for multiple comparisons), and between-system network interactions (β = 0.0001; 95% CI = 0.00,001, 0.00; *t*_(544)_ = 2.18; partial *r* = 0.09; *p* = 0.03, uncorrected), but not within-system network interactions (β = −0.0002; 95% CI = −0.001, 0.0001; *t*_(544)_ = −1.10; partial *r* = −0.05; *p* = 0.27), supporting the hypothesis that AD-related cognitive dysfunction is accompanied by alterations in brain network interactions that span multiple large-scale brain systems that are not limited to higher-order operations. As with the primary analyses, a participant's gender, head motion (i.e., postscrubbing mean FD), and years of education were included as covariates. Altogether, examination of the continuous measure of cognitive function reveals parallel relationships with system segregation and explains the unique variance relative to a participant's age, as was observed with CDR scores.

## Discussion

AD dementia severity and aging were independently associated with reductions in resting-state brain system segregation. Dementia severity-related brain network alterations were evident regardless of the presence of amyloid burden or AD-related genetic risk (presence of a copy of APOE4). Closer examination revealed that greater dementia severity and older age were associated with alterations in distinct sets of resting-state correlations, which were dissociable in terms of their functional roles and nature of network interactions. These results demonstrate that aging-related and dementia-related brain dysfunction can be untangled by examining alterations in large-scale resting-state network organization and that functional network organization can contribute a valuable source of information for AD characterization and staging.

### Alzheimer's disease dementia is associated with alterations in functional network interactions involving both association and sensory-motor systems

The present report dovetails with early reports implicating AD-associated alterations in relationships among default system regions (Greicius et al., 2004; Sorg et al., 2007; Binnewijzend et al., 2012; Damoiseaux et al., 2012; Badhwar et al., 2017), but demonstrates that AD dementia severity has more widespread effects on functional relationships that are not limited to either the default system or even association networks (Chhatwal et al., 2018; Fountain-Zaragoza et al., 2023). Rather, increasing dementia severity is associated with alterations in brain network relationships that involve systems implicated in both higher-order cognitive operations (association systems) and those involved in sensory and motor processing. These alterations are evident even in mild cases of impairment (i.e., CDR = 0.5), and are distinct from aging-related functional network alternations that tend to spare sensory-motor systems relative to association systems (Chan et al., 2014; Wig, 2017).

AD is classically associated with cognitive deficits in processes involving long-term memory and executive function (Salmon and Bondi, 2009; Weintraub et al., 2012). To this end, the observed alterations in sensory-motor network relationships may be unexpected (but for parallel observations see Strain et al., 2022). While appropriate data to make comparisons between brain network alterations and sensory and motor processing were not available in the present dataset, there is substantial evidence of early deficits in sensory processing among AD patients (Albers et al., 2015; Murphy, 2019). Compared with healthy individuals, individuals with MCI or advanced dementia can exhibit impairment in visual (Kirby et al., 2010), auditory (Loughrey et al., 2018), and olfactory processing (Wilson et al., 2009; Roberts et al., 2016). Olfactory impairment and hearing loss have also been reported to be preclinical markers for developing dementia symptoms in healthy adults (Wilson et al., 2009; Lin et al., 2011; Loughrey et al., 2018). Relatedly, both patients with MCI and dementia exhibit impairments in even rudimentary motor function (Kluger et al., 1997; Aggarwal et al., 2006; Zidan et al., 2012). Altogether, it is clear that sensory and motor function are not spared in AD dementia and that network alterations involving brain systems subserving sensory and motor processing may be early indicators of the disease.

### Interactions between functional systems are prone to dementia-related alterations

The deterioration of structural connections in AD patients has led to a “disconnection hypothesis” of AD dysfunction (Delbeuck et al., 2003; Stoub et al., 2006). An aspect of the present results is counterintuitive in this regard, whereby resting-state functional relationships across distinct brain systems are strengthened rather than weakened; earlier studies examining AD-associated alternations in resting-state network interactions did not report strengthening of between-system relationships (Brier et al., 2012; Chhatwal et al., 2018). A subsequent study demonstrated a relationship between increasing dementia severity and greater between-system RSFC correlations, although this relationship was specific to interactions between the default and frontoparietal task control systems (Contreras et al., 2019). Relatedly, AD-related brain network alterations have been reported using graph theory applied to several other brain-imaging techniques (Gong et al., 2009; Stam et al., 2009; Zhao et al., 2015; Kabbara et al., 2018; Cieri et al., 2021), and some of these observations conceptually align with the present resting-state BOLD findings. Nonetheless, the reason for the observed strengthening is presently uncertain, but it is possible that these changes are a maladaptive consequence of white matter structural disconnection (Grady et al., 2003; Dickerson and Sperling, 2008) and/or damage to brain network hubs. Hub regions facilitate information integration between distinct brain systems (Guimerà and Nunes Amaral, 2005; Power et al., 2013), and several reports have provided evidence that hub locations may be particularly prone to AD-related damage (Buckner et al., 2005; Drzezga et al., 2011; Crossley et al., 2014; Badhwar et al., 2017) and linked to the clinical manifestation of dementia (Roussarie et al., 2020).

Interestingly, descriptions of behavioral impairments in AD dementia converge with the idea that cross-network interactions may be particularly altered in the disease. Compared with healthy control subjects, AD dementia patients show lower performance in neuropsychological tasks involving integration of information across multiple sensory modalities (Salmon and Bondi, 2009; Vallet et al., 2013) but exhibit intact ability to process sensory inputs of each modality separately (Delbeuck et al., 2007). Notably, episodic memory impairments, a hallmark of AD, are largely due to disruptions in integrative or relational processing that are supported by medial temporal lobe structures (Cohen and Eichenbaum, 1993). The present brain network observations support the hypothesis that a primary deficit of AD dementia is that of information integration.

In contrast to dementia, age-associated alterations were prominent among within-system relationships. The weakening of within-system interactions observed with increased age is likely reflective of the progressive loss of brain area specialization (Park et al., 2004; Chan et al., 2017; Koen and Rugg, 2019). Previous work by our group and others have reported that increasing age is associated with both decreasing within-system relationships and increasing between-system correlations (Chan et al., 2014; Geerligs et al., 2015a; Grady et al., 2016). The latter was not evident in the present comparisons. It is possible that these previous reports of aging-associated alterations in between-system relationships were due to unaccounted preclinical pathology that may exist in individuals categorized as “healthy” (Brier et al., 2014b; Harrington et al., 2021). Alternatively, the discrepancy may relate to the degree of statistical control for cardiac and respiratory signals. These signals have been shown to confound age-related differences in functional correlations if appropriate steps are not taken to ensure mitigation of the sources of variance (Geerligs et al., 2017; Kong et al., 2020). However, the processing pipeline we used here is similar to that used in our previous work, which also demonstrated age-related alterations in between-system correlations (Chan et al., 2014), including a combination of techniques that both corrected the BOLD signal and covaried measures accounting for respiration-related and cardiac-related differences across participants. While both of the explanations above are plausible, one additional consideration is that the effects of age were estimated after adjusting for cognitive impairment scores across a range of healthy and unhealthy adults (both using CDR and using ADAS-Cog), which is different from previous reports that were conducted in largely healthy participant samples.

### Dementia-related alterations in brain system segregation are independent of the presence of amyloid pathology

Greater dementia severity is associated with lower brain system segregation, regardless of the presence of mean cortical amyloid burden. In addition, the spatial pattern of the observed functional network alterations is distinct from the distribution of AD-related brain pathology, where deposition of amyloid is most prominent in brain systems involved in memory and executive function operations but tends to spare sensory and motor systems, at least in early stages of the disease (Buckner et al., 2005; Jagust, 2013; Palmqvist et al., 2017). It remains to be seen whether the extent to which the functional network alterations reported here are more closely related to the presence of tau pathology (Steward et al., 2023) and whether they are specific to AD, especially as other forms of dementia typically impact unique sets of brain regions and are accompanied by distinct types of behavioral impairments (e.g., behavioral variant frontotemporal dementia, vascular dementia, Parkinson disease, atypical AD; Filippi et al., 2013, 2017; Gratton et al., 2019; Singh et al., 2023).

Importantly, the present results converge with several related observations which altogether motivate efforts to closely examine functional brain network organization in both preclinical and more advanced stages of AD. First, longitudinal changes in brain system segregation are prognostic of dementia independent of the presence of AD-related pathology among cognitively healthy individuals (cortical Aβ and CSF tau burden; Chan et al., 2021). Consistent with this, several other reports have also indicated the absence of a relationship between cortical Aβ and measures of system segregation in cognitively healthy individuals (Yang et al., 2022; Fountain-Zaragoza et al., 2023). Second, tau burden is associated with worse global cognition and episodic memory ability among AD patients, but this relationship is evident only for individuals exhibiting lower system segregation (Ewers et al., 2021). Relatedly, AD disease severity, measured as the estimated number of years to symptom onset among autosomal-dominant mutation carriers, is negatively related to brain system segregation (Ewers et al., 2021). The collective evidence indicates that alterations in resting-state brain system segregation operate on a path that is distinct from AD-related pathology and may be more closely related to AD-related cognitive dysfunction, thus providing a functional measure of cognitive resilience. Based on these observations, we recommend pursuing the incorporation of functional measures (*F*), such as resting-state system segregation into current AD biomarker frameworks [i.e., *AT*(*N*) – *F*; Jack et al., 2018].

### Limitations

There are several limitations of the current study that are important to acknowledge. First, we used cross-sectional data to compare the effects of adult aging and dementia. While these comparisons are informative toward understanding how the measures relate to one another, longitudinal analyses are needed to examine the changes of brain system segregation in relation to disease progression or healthy aging over time. Second, because individuals with more advanced dementia were more likely to be excluded because of poor data quality (e.g., they had a greater percentage of frames lost), individuals with higher CDR scores and more altered functional brain network organization may have been systematically excluded from the study. This possible selection bias could have resulted in underestimation of the relationship between greater dementia severity and lower brain system segregation. Last, the current study examines AD under the lens of its clinical manifestation. While we have incorporated available PET-based measures of amyloid deposition into our analysis (Fig. 1*D*), because of the imperfect agreement between clinical diagnoses and the presence of AD biomarkers (Cousins et al., 2021; Pascoal et al., 2023), it will be important to evaluate whether the observed dementia-related brain network alterations are specific to confirmed AD diagnosis.

### Conclusion

Although aging is a primary risk factor for AD, AD-related cognitive dysfunction and aging are associated with unique and dissociable patterns of alterations in large-scale functional brain networks. Our results indicate that the dementia-related brain network alterations are distinct from aggregate measures of AD-related amyloid pathology and may offer important clues and signals toward identifying the types of behavioral deficits that are most impacted at early stages of AD and other forms of dementia. Altogether, the current observations motivate refinement of functional network-based biomarkers of AD, which have the potential to contribute a unique source of information toward AD diagnosis and staging.
